# Integrated Photo‐Rechargeable Batteries: Configurations, Design Principles, and Energy Loss Mechanisms

**DOI:** 10.1002/smsc.202400598

**Published:** 2025-04-14

**Authors:** Tianyun Qiu, Wei Zhang, Xiaojing Hao, Kaiwen Sun

**Affiliations:** ^1^ School of Photovoltaic and Renewable Energy Engineering University of New South Wales (UNSW) Sydney NSW 2052 Australia; ^2^ Department of Chemistry University College London London WC1H 0AJ UK

**Keywords:** energy conversions, energy storages, photocharges, photo‐rechargeable batteries, photovoltaics

## Abstract

Integrated photo‐rechargeable batteries (IPRBs) represent an emerging device class that enables simultaneous energy conversion and storage, opening new possibilities for sustainable self‐powered energy solutions. The rapid advancements in this ascendant field have led to multitudinous constructions and designs, each differing in charge storage mechanisms and carrier dynamics. In this review, these works are revisited and classified into three main types: the photoelectrochemical batteries, the all‐in‐one monolithic IPRBs, and the photovoltaic–battery integration, which can be further categorized by their electrochemical configurations and working principles into two‐terminal, three‐terminal, and four‐terminal architectures. This study delves into the common issue of IPRBs, namely their energy loss mechanisms, offering a comprehensive overview of current research progress, challenges, and future research directions. This review aims to provide insights and rational guidelines for designing the next‐generation high‐performance IPRBs.

## Graphical abstract

This review explores integrated photo‐rechargeable battery architectures that unify solar energy conversion with electrochemical storage. By analyzing two‐terminal, three‐terminal, and four‐terminal designs, this study reveals how the working mechanisms, materials, and energy loss pathways govern efficiency and propose design strategies. These insights provide a roadmap for next‐generation, self‐powered systems with enhanced stability and lower environmental impact.

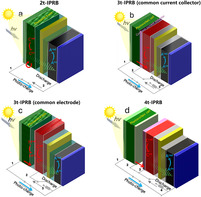

## 1 Introduction

Solar photovoltaic (PV) technology, known for its nonpolluting attributes and reliance on abundant solar irradiation, plays an important role in the renewable energy transition. However, despite advancements that have yielded impressive power conversion efficiencies—such as up to 47.6% in concentrated four‐junction PV cells and 26.7% in single‐junction silicon cells—PV technology is inherently limited by the intermittency and variability of solar irradiance.^[^ ^1^, ^2^ ^]^ Solar output fluctuates over daily and seasonal cycles, as well as in response to weather patterns, presenting challenges for consistent energy supply. This variability hinders PV's potential as a reliable, standalone energy source.

Integrated photo‐rechargeable batteries (IPRBs) are an emerging class of energy storage technologies that integrate solar energy conversion and electrochemical storage into a single, compact device. Among various hybrid solar harvesting and storage systems—which encompass a wide range of technologies such as photosupercapacitors, PV‐electrochemical hybrids, and solar fuel generators—IPRBs stand out for their unique ability to directly store solar energy in a rechargeable battery, offering distinct advantages in addressing solar intermittency and ensuring stable power output. These systems allow for energy capture when sunlight is available and convert it for use during periods when sunlight is absent.^[^ ^3^, ^4^ ^]^ By providing a continuous power supply, IPRB systems reduce dependence on conventional energy sources and enhance the resilience of off‐grid and autonomous self‐powered applications, making them a crucial part of reducing carbon emissions across a wide range of scenarios.^[^ ^5^, ^6^ ^]^

One key advantage of IPRBs is their capacity to balance the intermittency of solar power by storing and releasing energy as needed—charging during sunlight hours and discharging when sunlight is insufficient or absent. This capability enables smoother, more stable power output, making IPRBs valuable for both on‐grid and off‐grid applications.^[^ ^7^, ^8^ ^]^ For on‐grid systems, IPRBs help balance power fluctuations, bolster grid resilience, and provide a reliable backup during supply disruptions. In off‐grid scenarios, they supply dependable power for next‐generation devices.^[^ ^9^ ^]^ By consolidating solar energy conversion and electrochemical storage into an integrated device, IPRBs offer streamlined efficiency for applications in remote sensing, environmental monitoring, wildlife tracking, and precision agriculture.^[^ ^10^, ^11^, ^12^, ^13^ ^]^ Additionally, IPRBs are promising for autonomous power supply in applications like Internet of Things (IoT) devices and energy‐independent systems across diverse sectors, including electric vehicles and aerospace technology, where continuous power is required while the grid is absent.^[^ ^14^, ^15^, ^16^, ^17^, ^18^ ^]^

While IPRBs offer remarkable advantages, several challenges remain, particularly in optimizing charge separation to storage efficiency, enhancing interfacial charge transfer kinetics, and ensuring long‐term cyclic stability. Advancements in materials science and fabrication methodologies are anticipated to further improve the performance of IPRBs, broadening their applications. As an emerging photo‐to‐electrochemical energy storage technology, IPRBs play a crucial role in advancing next‐generation devices, contributing to a resilient and sustainable energy future.

**Figure 1:**
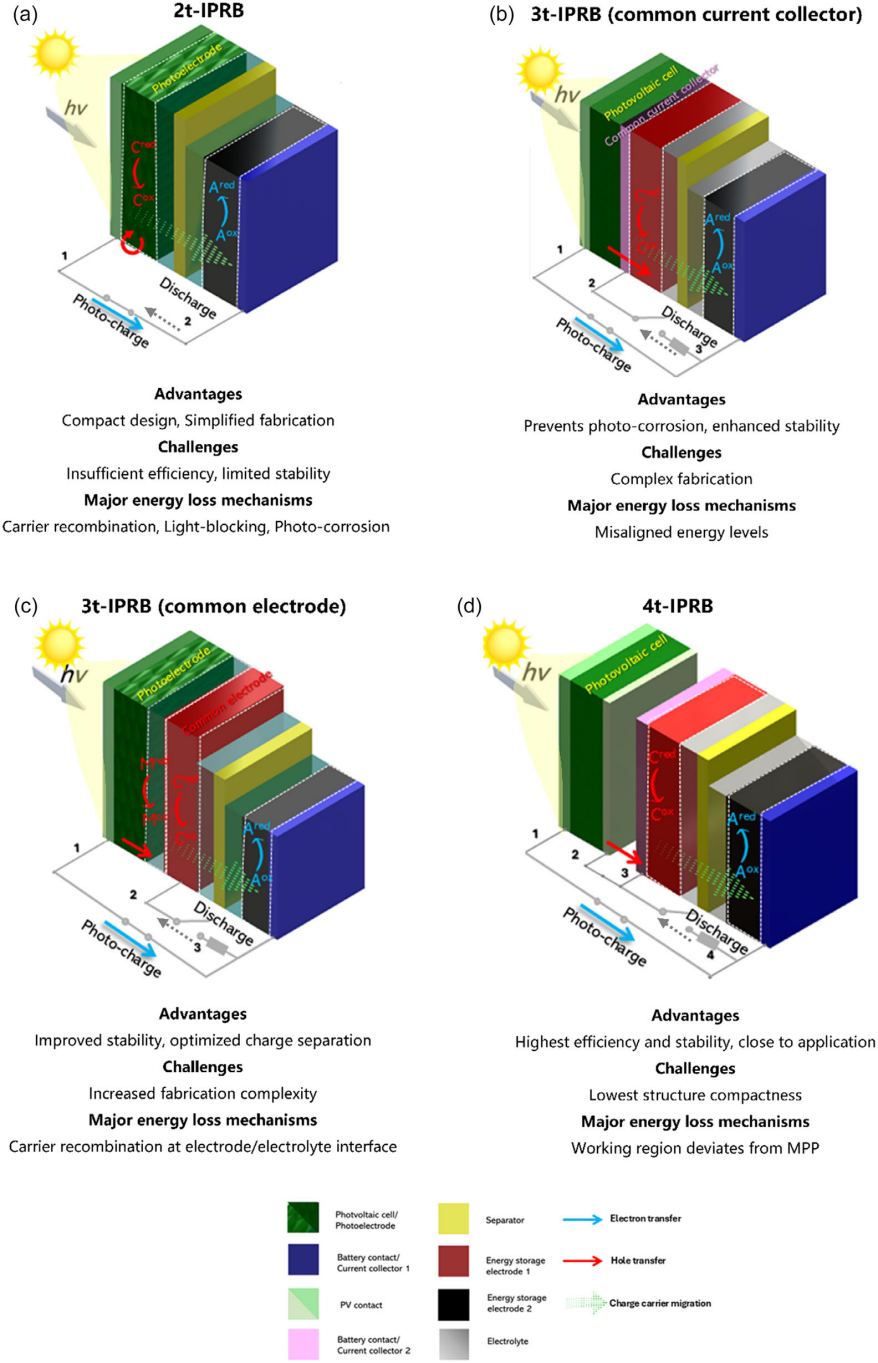
Schematics illustration on different IPRB configurations under photocharging working conditions. The closed circuit represents the photocharging condition, while the open circuit shifts to closed during dark discharge. The annotations A, C, and M denote the anode, cathode, and redox mediator respectively. a) 2t‐IPRB. b) Common current collector 3t‐IPRB. c) Common electrode 3t‐IPRB. d) 4t‐IPRBs. The green textured pattern represents the photoresponsive materials mixed with other materials, for example, binder, conductive.

In this review, we classify the IPRBs into the following categories according to their electrochemical traits and their operation characteristics (**Figure** **1**): two‐terminal IPRBs (2t‐IPRBs) constituted by a bifunctional photoelectrode which take place in the photoelectrochemical reaction, three‐terminal IPRBs (3t‐IPRBs) with monolithic architecture enabled by a shared mediator, and the direct coupled four‐terminal IPRBs (4t‐IPRBs). This review will examine and summarize the key milestones in the development of three types of IPRBs and then delve into their principles, architectures, strengths, and drawbacks.

The first cornerstone of 2t‐IPRB research was reported by Hodes et al. in 1976; they demonstrated a polycrystalline chalcogenide cadmium selenide photoelectrode.^[^ ^19^ ^]^ The photogenerated electrons and holes are separated due to the built‐in electric field, and then the electrons move to the external circuit, while the holes migrate to the electrode surface, participating in the redox reaction enabled by S^2−^/S redox mediators in the electrolyte. The photocurrent and photovoltage thus have been generated and maintained, and the energy storage was driven by the redox couple Ag/Ag_2_S, where silver has been reversibly oxidized and reduced to release and store electrons. In this work, the 3t‐IPRB configuration has also been tested by separating the storage electrode and counter electrode, and the 3t‐IPRB design increases the storage current efficiency to ≈90%. This work elucidates the potential of photoelectrochemical cells (PECs) for solar energy conversion and storage, validating the foundational principles for later‐on IPRB research and designs. In 2t‐IPRB configuration, as shown in Figure 1a, a bifunctional photoelectrode fulfills dual roles—serving both as the light‐harvesting element and as the energy storage component. Upon light absorption, the photoelectrode initiates photoinduced electrochemical reactions, resulting in the simultaneous generation and storage of electrical energy within the same material. Conductive additives and binders are incorporated into the photoactive materials to enhance electrical conductivity and stability, at the expense of reduced light absorption. Under illumination, when the photon energy surpasses the bandgap of the solar cell absorber, the electron transport from the valence band to the conduction band and then transferred to the electrode during the photocharging. The process happens accompanied by ion diffusion and phase transition within the electrode material. The simultaneous storage and photoconversion reactions create a complex interplay between ion intercalation and electronic charge carriers. The dual functionality leads to challenges such as phase variation during cycling, charge recombination, and light absorption losses. Additionally, the photo corrosion of electrodes further exacerbates these issues, limiting overall performance.

Following the 2t‐IPRB, early research on 3t‐IPRB was reported in 2004. The 3t‐IPRBs design aims for the avoidance of photointercalation and phase transitions of photoelectrode during charge/discharge.^[^ ^20^ ^]^ This work developed a dye‐sensitized TiO_2_ as the photoanode, absorbing photons and generating photoexcited dye molecules, polypyrrole. The polypyrrole played the role of energy storage electrode, gaining the photogenerated electrons from the photoanode and facilitating the redox reactions in the electrolyte. A platinum counter electrode was applied to balance the oxidation reactions occurring at the photoanode. The I^−^/I_3_^−^ redox shuttle in the electrolyte serves as the redox mediator, building a pathway for electron transfer between the photoanode and the counter electrode. The transition from 2t‐IPRBs to 3t‐IPRBs marks an enhancement in terms of structural stability and electron‐hole separation.

The 3t‐IPRBs have emerged as a promising paradigm to address the limitations of conventional 2t‐IPRB architectures, where the photoelectrode's direct exposure to the electrolyte often leads to photocorrosion, reduced cycling stability, and suboptimal energy‐conversion efficiency. By introducing a shared component that mediates photoelectric conversion and electrochemical energy storage, 3t‐IPRBs effectively decouple these processes, thereby offering enhanced operational voltages, improved stability, and higher overall efficiency compared to traditional 2t systems.

According to the function of shared component, the 3t‐IPRBs can be categorized into common current collector‐based and common electrode‐based configurations (Figure 1b,c). In the common current collector‐based design, a conductive substrate protects the photoelectrode from the electrolyte while maintaining photovoltage and facilitating charge transport. Conversely, the common electrode‐based configuration features an electrode that actively engages in redox processes—often involving shuttle species such as I^−^/I_3_^−^ or S^2^^−^/S_x_^2^^−^—thereby mitigating large overpotentials and enabling complex electrochemistry. Such an approach can mitigate large overpotentials and facilitate multistep electrochemical processes—particularly advantageous in applications such as lithium–sulfur or zinc–air batteries.

Further details regarding the mechanisms, material considerations, and electrochemical performance of these two 3t‐IPRB configurations are discussed in Section 3. By tailoring the shared component's structure and function, 3t‐IPRBs offer promising routes to more robust, efficient, and versatile solar‐energy storage.

Compared to the 2t‐ and 3t‐IPRBs, the 4t‐IPRB presents a promising solution for direct coupling of a battery with a PV module at a module scale. In this configuration, the battery itself plays the role of power and impedance coupling component. The integrated module is able to demonstrate a high degree of energy coupling, surpassing systems that even utilize maximum power point tracking (MPPT), through precise optimization of the impedance and voltage matching between the PV modules and the battery.^[^ ^14^, ^15^ ^]^ By minimizing the reliance on complex electronic management systems and optimizing the charging dynamics to adapt to fluctuating light conditions, the direct coupling effectively mitigates the inefficiencies commonly encountered in IPRBs, offering a streamlined and efficient alternative for integrated energy systems. The proof‐of‐concept work on 4t‐IPRBs was reported in 2010 by Kelly et al.^[^ ^21^ ^]^ The system demonstrated a direct connection between the commercial silicon PV modules and commercial lithium–ion batteries (LIBs) modules without additional intervening electronics. 15 LIBs were wired in series and charged at a rate of up to 1.5 C, fully charged in ≈40 min, yielding an overall system efficiency of 14.5%. This high efficiency was achieved by aligning the MPP of the PV module with the charging voltage of the battery. Different from the previous two IPRBs, the standalone working principle of 4t‐IPRBs (Figure 1d) enables independent optimization of PV and energy storage components to their best performance. This decoupled design is particularly effective in minimizing interdependent losses due to interdependencies, achieving a highest degree of energy coupling by minimizing impedance mismatches between the PV module and the battery. It also alleviates the issues in other types of IPRBs, such as photoelectrode degradation and impedance mismatch. As a consequence, this configuration results in higher overall system efficiency and reduced energy losses due to interdependent issues. For direct 4t‐IPRBs, the overall efficiency could be expressed as

(1) $$\begin{array}{c} \eta_{\text{overall}}=\eta_{\text{PV}‐\text{battery }}\cdot \eta_{\text{round trip}} \end{array}$$

$$n_{\text{round trip}}=\frac{\int_{t_{\text{d,start}}}^{t_{\text{d,end}}}P_{\text{out}}dt}{\int_{t_{\text{c,start}}}^{t_{\text{c,end}}}P_{\text{in }}dt}$$

The roundtrip efficiency is determined by the ratio of the discharge output energy to the input charge energy. This includes possible Joule heat generation, Coulombic losses, overpotential, polarization, and resistive losses in the battery. In conventional PV–battery integration techniques, the reliance on electronic management devices, such as MPPT units and inverters, can introduce additional energy losses during transmission and conversion. These losses are often due to inefficient impedance matching and resistive losses. In contrast, 4t‐IPRBs minimize these losses by directly coupling the PV module with the battery, thereby eliminating the need for such complex electronics and enabling more efficient energy transfer. As for the 3t‐ and 2t‐IPRBs, the conversion efficiency is not directly attained from solar cells, and the overall efficiency is described as

(2) $$\begin{array}{c} \eta_{\text{overall}}=\frac{E_{\text{output}}}{P_{\text{in}}\times A\times t} \end{array}$$

where *E*_output_ refers to the total energy discharged from the battery, *P*_in_ refers to the input power density on the photoelectrode, and *A* represents the effective area under illumination. Although 2t‐ and 3t‐IPRBs have achieved reduced volumes and higher compactness, these advancements come at the cost of overall efficiency. Specifically, the 4t‐IPRBs exhibit a higher overall efficiency (up to 23.11%) compared with monolithic 3t‐IPRBs and 2t‐IPRBs (up to 5.14%),^[^ ^22^, ^23^ ^]^ which is ascribed to the standalone working principles. The decoupled working principle ensures the optimization of each component independently, circumvents the parasite reaction such as photo electrolysis, and avoids charge/hole recombination at the interface to the maximum extent. Regarding 2t‐IPRBs, the photoelectrode faces a trade‐off between achieving high power conversion efficiency and maintaining energy storage efficiency.

As an emerging research area, the IPRBs are still at an early stage of research but have garnered increasing interest. However, there is still a gap in the understanding of photocharging behaviors and general design principles. Herein, this work aims to address the fundamental concepts underlying the IPRBs by providing a comprehensive review of the state‐of‐the‐art research on material science and electrochemical characteristics. It analyzes how the architecture of the three types of IPRBs influences the overall operational performance. Through comprehensive detailed insights, this review strives to guide future design strategies for highly efficient, stable devices while promoting innovation in the practical implementation of IPRBs in sustainable energy applications.

## 2 2t‐IPRBs

The 2t‐IPRBs offer a compact and efficient system composed of a dual‐functional photoresponsive energy storage electrode and a counter electrode. The dual‐functional electrode plays a crucial role because it participates in both photoelectric conversion and energy storage.

### 2.1 Photoelectrode Materials and Working Mechanisms

As the core component of 2t‐IPRBs, the bifunctional photoelectrode consists of a semiconducting material that serves as the active component. Considering the stability against irradiation corrosion and efficient charge separation and transport, the high‐bandgap semiconductors such as TiO_2_ (E*g* ≈ 3.2 eV) and SnO_2_ (E*g* ≈ 3.6 eV) are utilized as photoelectrode material, despite it at expense of limited adsorption of the solar spectrum (<5%).

Another emerging type of material is layered transition metal compounds from Group IV and Group V, such as dichalcogenides (TX_2_ with T = Mo, W, Zr, Hf, X = S, Se) and layered transitional metal oxides.^[^ ^24^, ^25^ ^]^ The layered architecture accommodates ions (Li^+^, Na^+^, and Zn^2+^) to facilitate reversible ion intercalation without significant structural degradation during photocharge.

#### 2.1.1 Photon Absorption and Charge Separation

Typically, the photocharge process of 2t‐IPRB starts from the photon absorption and charge separation stage. When the photoelectrode is illuminated with light of photon energy (*h* *v*) exceeding its bandgap (E*g*), electron‐hole pairs are generated.

(3) $$Semiconductor\left(h\nu \right)\to e^{-}+h^{+}$$

The built‐in electric field at the depletion region drives the photogenerated electrons (the majority carriers in N‐type semiconductor) toward the bulk of the semiconductor, while the holes (the minor carriers in N‐type semiconductor) migrate toward the surface then drive the oxidation reactions of photoelectrode, when the electrons travel through the external circuit to the counter electrode and reduce the metal ions on the counter electrode.
(4)

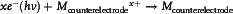




#### 2.1.2 Energy Storage

The energy storage mechanisms of 2t‐IPRBs vary depending on the physical properties of the materials and the pathways through which the photogenerated carriers are utilized. Here, we classified them into two types.

##### Photoinduced Oxidation

During the charge separation process, the photogenerated holes accumulate at the valence band of the photoelectrode surface and migrate toward reaction regions, and then the self‐oxidation is triggered, generating the oxidized state of the active material. The photovoltage magnitude generated by illuminating the space charge layer of the semiconductor junction depends on the bandgap and counter electrode redox potential. To be specific, when the photovoltage is high enough to drive the counter electrode redox potential, no bias voltage is required; otherwise, it turns to the photo‐assisted charging mode.

To improve the hole's mobility and utilizations, materials with overlapping *p*‐orbitals (e.g., transition metal oxides like TiO_2_ and α‐Fe_2_O_3_) are applied, as holes could hop between adjacent oxygen atoms via their overlapping orbital.^[^ ^26^ ^]^ Undoubtedly, the photoinduced self‐oxidation mechanisms enable its application in self‐charging energy storage fields, yet still, this type of material suffers from several drawbacks, such as limited spectral utilization, high carrier recombination, low electrical conductivity, and low stability. To address these issues, researchers have developed strategies including heterogeneous structure engineering that combines the semiconductive material with other conductive additives, catalysts, passive layers, and different semiconductors that enhance the band alignment.^[^ ^8^, ^27^ ^]^

The photoinduced oxidation mechanisms have been discovered and widely applied in battery fields. For instance, in photo‐assisted Li–O_2_ battery works, the TiO_2_ as an additive of photoanode generated holes, which can not only reduce the charge potential, but also leave abundant oxygen vacancies to enhance the electron transfer.^[^ ^28^ ^]^ Another Li–O_2_ battery work applied Fe_2_O_3_–TiO_2_ as bifunctional photoelectrodes and stated that the photogenerated hole could reduce the reaction overpotential and accelerate the decomposition of Li_2_O_2_.^[^ ^29^ ^]^ Besides the application of metal–air batteries, another study demonstrated a CdS‐TiO_2_ heterostructured cathode in Li–S battery, where the photogenerates holes (under illumination) accelerate the sulfur evolution reaction and facilitate the oxidation of Li_2_S to lithium polysulfides.^[^ ^30^ ^]^ To conclude, the photoinduced oxidation mechanisms greatly reduce the reaction energy barrier and polarization and accelerate the kinetics of electrode chemistry especially in multielectron transfer reactions (metal–air, metal–sulfur) in battery applications.

##### Photointercalation

Differ from the photoinduced oxidation mechanism, photointercalation typically involves the photogenerated electrons dominating step in energy storage. To accommodate the ion insertion/extraction, these materials possess with layered crystal structures, enabling the incorporation of photogenerated electrons and ions into their lattice. When charge separation, photogenerated electrons migrate through the conduction band of the photoelectrode toward the interface and are inserted into the host material, which induces the reduction reaction. Simultaneously, the charge carrier cations M^a+^ (A = Li^+^, Na^+^, K^+^, Zn^2+^) from the electrolyte intercalate into the lattice to form alkali compound.

**Figure 2:**
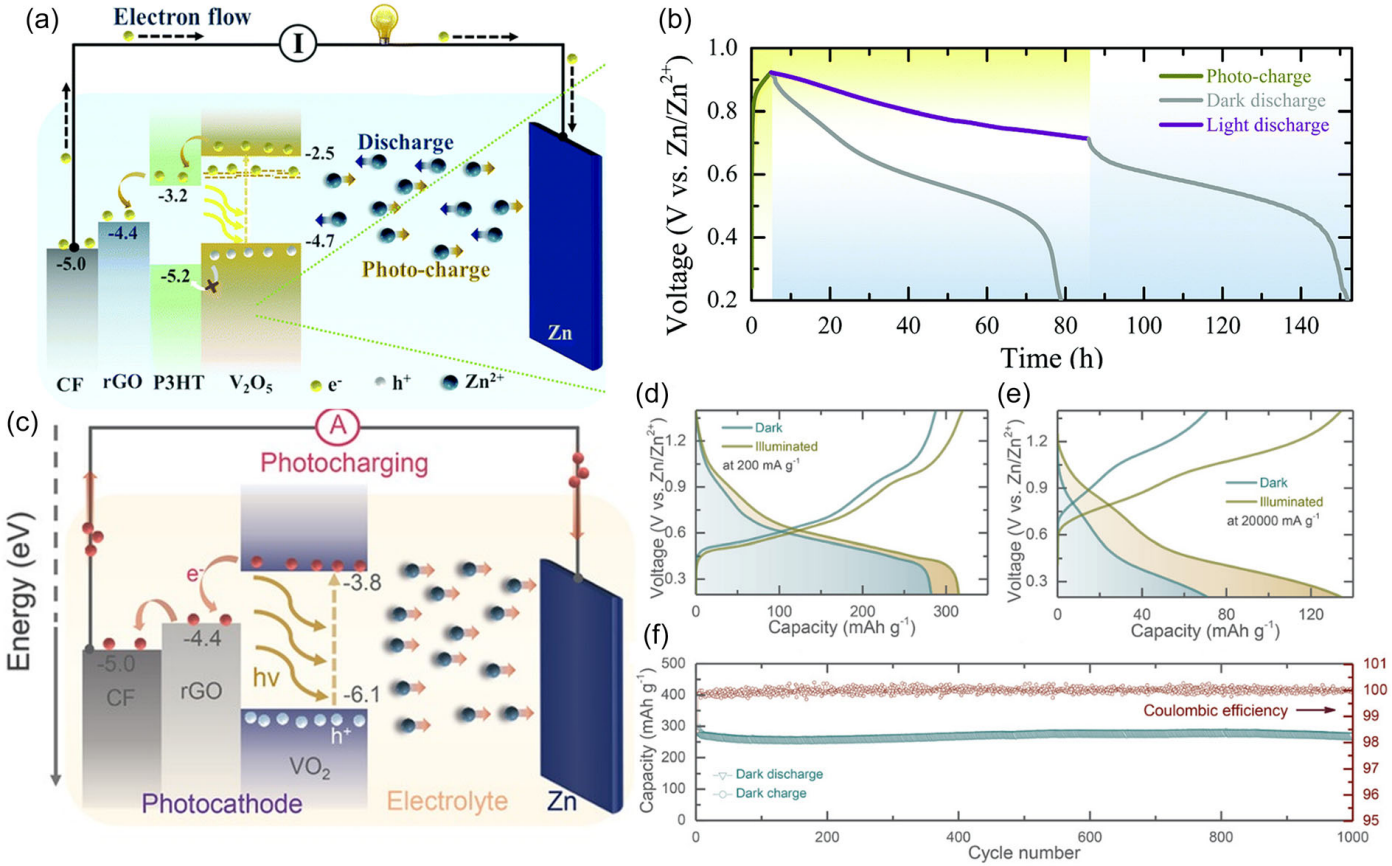
a) Illustration of the photocharging mechanism of two‐electrode photo‐ZIBs. b) Photocharge (λ ≈ 455 nm, intensity ≈12 mW cm^−2^) and constant current discharge of the photo‐ZIB (specific current of 100 mA cm^−2^) in dark and illuminated conditions. Reproduced with permission.^[^ ^32^ ^]^ Copyright 2020, Royal Society of Chemistry. c) Illustration of the proposed photo charging mechanism of two‐electrode VO_2_–rGO photo‐ZIBs. d) Galvanostatic discharge–charge curves at specific currents of 0.2 A g^−1^ in dark and illuminated conditions e) Galvanostatic discharge–charge curves at specific currents of 20 A g^−1^ in dark and illuminated conditions. f) Long‐term cycling test of the two‐electrode photo‐ZIB at a specific current of 1 A g^−1^ in dark. Reproduced with permission.^[^ ^33^ ^]^ Copyright 2021, Wiley‐VCH.

A 2t‐photo‐rechargeable lithium ion battery applied TiS_2_–TiO_2_ as a cathode has been presented by Narayanan et al.^[^ ^31^ ^]^ In this system, the type‐II semiconductor heterojunction between TiS_2_ and TiO_2_, which is characterized by staggered energy band alignment where the conduction band of TiS_2_ is lower and the valence band of TiO_2_ is higher, facilitates efficient charge separation by directing electrons and holes into different materials. The transferred electrons reduce the TiS_2_ lattice (Ti^4+^ → Ti^3+^), creating localized sites for lithium‐ion intercalation from the electrolyte into the layered structure of TiS_2_. This photointercalation process directly converts solar energy into stored chemical energy within the battery. Moreover, the layered structure of TiS_2_ and its high lithium‐binding energy ensures effective lithium‐ion photointercalation, while the synergy between TiO_2_ and TiS_2_ ensures efficient light harvesting and charge transfer. This design highlights a novel integration of solar energy harvesting and lithium‐ion storage, positioning this system as a promising solution for next‐generation photo‐rechargeable batteries. As can be seen in **Figure** **2**a, a photo‐rechargeable two‐electrode zinc‐ion batteries (ZIBs) applying V_2_O_5_ nanofibers as the dual‐functional electrode was proposed by Volder et al.^[^ ^32^ ^]^ By integrating reduced graphene oxide (rGO) and poly(3‐hexylthiophene‐2,5‐diyl) (P3HT), the V_2_O_5_ nanofibers effectively generate and separate electron‐hole pairs under visible light illumination, facilitating Zn^2^^+^ ion intercalation/de‐intercalation. The system achieved a remarkable increase in capacity from ≈190 mAh g^−1^ in the dark to ≈370 mAh g^−1^ under illumination, with a photoconversion efficiency of ≈1.2% (Figure 2b). The introduced rGO and P3HT in the systems act as carrier separation components, building a well‐matched energy level structure. In this configuration, rGO acts as an electron transfer scaffold, while P3HT primarily functions as a hole‐blocking material, aligning with the energy level diagram shown in Figure 2a. This energy alignment hinders hole transfer to adjacent layers, thereby reducing recombination losses and enhancing charge separation efficiency. Apart from this work, ZIBs, using rGO/VO_2_ photocathodes have been reported by the same group.^[^ ^33^ ^]^ In this design, the energy‐level alignment between carbon fiber, rGO, and VO_2_ enables efficient electron transport, reducing recombination losses and enhancing charge separation. Furthermore, the incorporation of rGO establishes a favorable energy pathway for separating photogenerated electrons and holes. This bias‐free operation under illumination underscores the potential of VO_2_/rGO/carbon‐fiber configurations for advanced photo‐rechargeable ZIBs, as depicted in the energy diagram in Figure 2c. Compared to the former work, it achieved approximately doubled capacity, with a capacity retention of ≈90% over 1000 cycles (Figure 2f). TiO_2_@MoS_2_@N‐doped carbon heterostructure photoelectrode was reported by Chen et al.^[^ ^34^ ^]^ The TiO_2_@MoS_2_ heterostructure employs a direct Z‐scheme mechanism, where the conduction band of MoS_2_ aligns below the valence band of TiO_2_, creating a built‐in electric field at the interface that enhances charge separation. During discharge, MoS_2_ undergoes a two‐step conversion reaction to form Mo and Na_2_S. Under illumination, photogenerated holes in the valence band of MoS_2_ recombine with electrons in the conduction band of TiO_2_, while the remaining charge carriers—electrons in the conduction band of MoS_2_ and holes in the valence band of TiO_2_—drive sodium storage reactions. This Z‐scheme mechanism ensures spatial separation of photogenerated carriers after selective recombination, reducing overall carrier losses and enhancing reaction kinetics. An N‐doped carbon coating further improves conductivity, achieving a photoconversion efficiency of 0.71% under illumination.

It is noteworthy that in the photocharge regime, the impedance of the photoelectrode can be greatly reduced, regardless of the state of charge (SoC) of the battery. This phenomenon could result from the increase in charge carrier density, improved charge separation and passivation of surface states.^[^ ^35^, ^36^, ^37^, ^38^ ^]^

It could be concluded that although the two‐electrode configuration shows the highest compactness in structure, it still faces challenges in enhancing photoconversion efficiency. On the one hand, the low efficiency is caused by the insufficient light absorption of the photoelectrode as the photoresponsive materials (V_2_O_5_, VO_2_, TiO_2_, and MoS_2_) are mixed with binder and conductive during the electrode fabrication process. On the other hand, the photo(de)intercalation of photoelectrode alters its optical properties and band structure, leading to suboptimal charging characteristics.^[^ ^39^, ^40^ ^]^ Furthermore, the intrinsic thermodynamic factor in dual‐functional electrodes is inevitable, which is the carrier lifetime mismatch between photogenerated charges (≈μs) and Faradic reaction (≈ms), resulting in a massive waste of carriers.^[^ ^8^ ^]^

## 3 3t‐IPRBs

To address the issues identified in 2t‐IPRBs, the 3t‐IPRBs are proposed. The 3t‐IPRBs combine the advantages of 2t‐IPRBs and 4t‐IPRBs with a compact monolithic structure that requires no additional connections. This cohesive architecture is enabled by either a common current collector or a shared electrode between photo‐harvesting electrode and energy storage electrode. This rational design not only protects the photoresponsive electrode from photocorrosion by the electrolyte but also prevents phase transition caused by photoelectrochemical reactions.^[^ ^41^, ^42^ ^]^ Multitudes of works have been reported on 3t‐IPRBs. Here, we clarify the principle and the functionality of the materials in each component, categorizing them into two main types: common current collector 3t‐IPRBs and common electrode 3t‐IPRBs.

### 3.1 Common Current Collector 3t‐IPRBs

In this configuration, the common current collector serves as an intermediary between the photoelectrode and the energy storage electrode, ensuring efficient carrier transport while preventing direct contact between the photoelectrode and the electrolyte. By minimizing recombination losses and stabilizing the photovoltage, the current collector enhances energy conversion efficiency.

Because it remains largely inert electrochemically, the current collector does not engage in Faradaic reactions but instead serves to shuttle photogenerated carriers into the storage electrode. To achieve optimal performance, the collector material must exhibit high electrical conductivity (reducing resistive losses), chemical stability (resisting electrolyte degradation), and appropriate energy‐level alignment with the photoelectrode. Materials such as conductive carbon paper, metallic foils (e.g., Al and Cu), and engineered composites have successfully been used for this purpose. When combined with advanced PV technologies (e.g., silicon, perovskite, and organic cells) and various metal‐ion batteries (e.g., Li–ion, Na–ion, and Zn–ion), these designs show markedly improved long‐term stability compared to conventional 2t‐IPRBs.

#### 3.1.1 ZIBs‐Based IPRBs

Numerous studies have demonstrated the ZIBs exhibits great cycling performance at high current density (≥5C). It is primarily due to capacitive‐dominated capacity contribution, rather than the conventional diffusion‐controlled battery behavior.^[^ ^43^, ^44^ ^]^ In capacitive‐dominant electrode, the wide slope region during charge and discharge indicates that there is no minimum charging voltage requirements. The battery capacity is correlated to the photovoltage generated by photoelectrode. The higher open‐circuit voltage (V_oc_) from photoelectrode delivers larger capacity to ZIBs electrode, as long as the cutoff voltage is within the electrochemical window to avoid the electrolyte hydrolysis. As a result, the deficiency in V_oc_ may still charge the ZIB but could drive the ZIB to photo‐assisted charging regime, requiring a bias to completely charge the battery. These properties enable ZIBs to couple with single‐junction solar cells, which deliver voltage within its operation range. In addition, the aqueous electrolytes in ZIBs endows its nonflammable characteristics, thereby ensuring the safety of IPRBs

**Figure 3:**
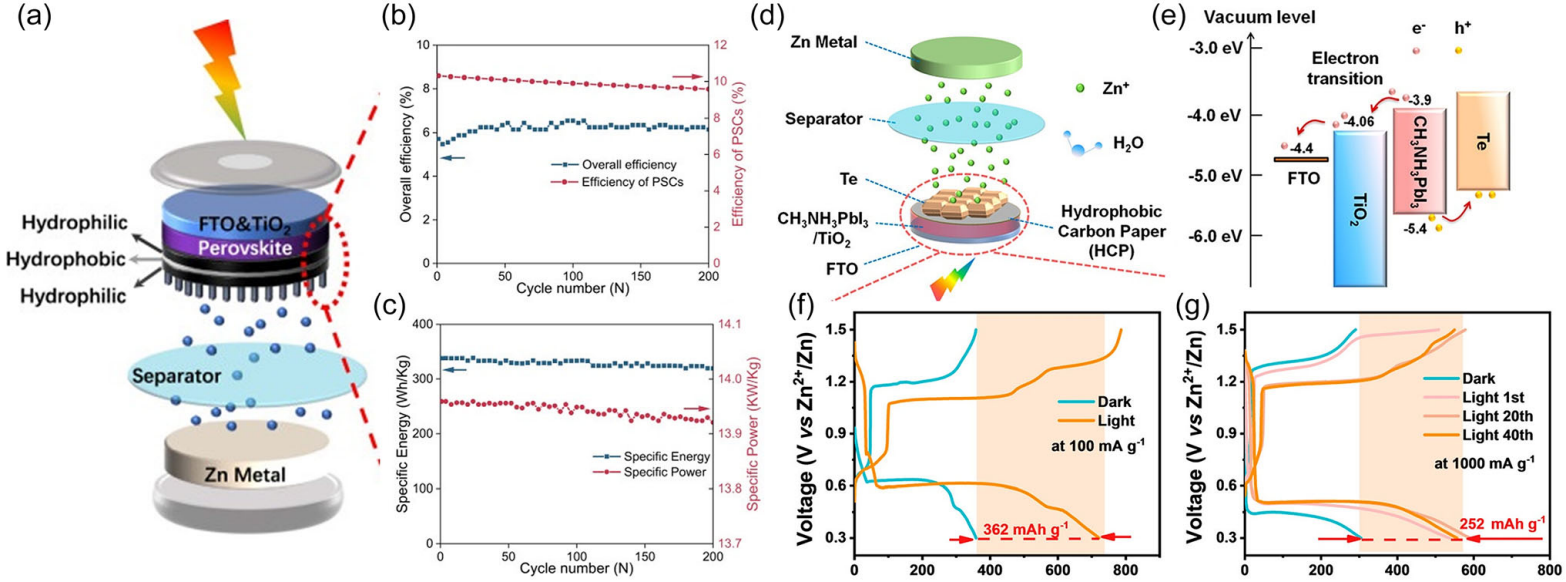
a) Schematic representation of the 3t‐photo‐rechargeable zinc–ion battery. b) Power conversion efficiency of the module and overall efficiency during 200 photocharge and discharge cycles (0.42 cm^2^ and 8 A g^−1^). c) Specific energy and power performance during 200 photocharge and discharge cycles. Reproduced with permission.^[^ ^45^ ^]^ Copyright 2022, Springer Nature. d) Schematic representation of the 3t‐photo‐rechargeable Zn–Te battery, e) corresponding energy band diagram. Electrochemical performances comparison for the Zn–Te battery in light and dark conditions at f) 100 mA g^−1^ and g) 1000 mA g^−1^. Reproduced with permission.^[^ ^44^ ^]^ Copyright 2023, American Chemical Society.

Perovskite solar cell emerged as a promising candidate for ZIB‐based 3t‐IPRBs, as it demonstrated relatively high conversion efficiency and V_oc_, which can charge the battery without bias voltage. The flexibility in fabrication also makes it able to incorporate with energy storage electrode monolithically or even tandem to further increase the V_oc_. In 2022, Gao et al. reported integrated solar rechargeable ZIBs enabled by perovskite.^[^ ^45^ ^]^ This device integrates an aqueous zinc battery with a hole transport layer‐free carbon‐based perovskite module. Apart from this, as shown in **Figure** **3**a, the heterostructural Co_2_P–CoP–NiCoO_2_ nanoneedle arrays are in situ fabricated on one hydrophilic side of the sandwich joint electrode, and the perovskite light absorber was fabricated on the other side with an illumination window on the top. The hydrophilic–hydrophobic–hydrophilic‐structured carbon paper plays the role of a common current collector, accepting the photogenerated holes and driving the oxidation of the P–NiCo_2_O_4_ electrode. The hydrophilicity on the external surface ensures the battery electrochemical activity and wettability, and the hydrophobicity inside effectively prevents the perovskite from electrolyte penetration. The device demonstrates high performance with specific energy of 366 Wh kg^−1^ and specific power of 54.01 kW kg^−1^, along with an overall efficiency of 6.4% (Figure 3b,c). Another photo‐rechargeable aqueous zinc−tellurium battery utilizing a Janus‐jointed photocathode was reported by Li et al. in 2023.^[^ ^44^ ^]^ The well‐matched energy levels within the Te/CH_3_NH_3_PbI_3_/TiO_2_ Janus‐jointed photocathode ensure efficient conversion of photo energy into electrical energy by facilitating charge transfer between the photoexcited components, as shown in Figure 3d. A hydrophobic carbon paper acts as a barrier and mediator, maintaining the photovoltage and drive the alloying reaction of Zn–Te battery. As in Figure 3e, the design leverages the complementary energy levels of perovskite and tellurium to enhance charge transfer efficiency, enabling effective photo charging capabilities. The device showed an overall efficiency of 12% and the photoconversion efficiency of 0.31%. It can be seen from Figure 3f and Figure 3g that the charge capacity increased from 253 to 655 mAh g^−1^ at 1000 mA g^−1^ under light illumination. The additional capacity originates from the capacitance contribution of the CH_3_NH_3_PbI_3_, where the photogenerated carriers stored on the surface contributes electric double‐layer capacitance. It indicates the possibility that light illumination not only triggers the redox reaction but also shape the electrochemical traits of battery in 3t‐IPRBs.

#### 3.1.2 LIBs‐Based IPRBs

Different from ZIBs, the LIBs exhibit a Faradic storage behavior. It is represented by a plateau region in charge and discharge graph or quasi‐square‐shaped cyclic voltammetry.^[^ ^46^ ^]^ The flat plateau region indicates potential of the redox reaction or phase transition occurring. As a result, it reflects that a minimum charge voltage input is required to overcome the redox reaction potential. Photovoltage lower than that number would end up being a photo‐assisted charging regime, which requires a bias voltage. The LIB plateau potential is dependent on the electrodes and charge/discharge current density, typically ranging from 3.2 to 4.2 V. Series‐connected solar panels or multijunction solar cells are applied in LIBs‐based IPRBs to meet this standard. The merits of Faradic capacity batteries lie in the steady voltage and stable operating voltage, which is crucial for IPRBs performance and safety. Efforts have been made on organic solar cells and silicon solar cells in monolithic 3t‐IPRBs; for the former one, the solution‐based fabrication process makes the multijunction fabrication possible, and the latter is stable in various conditions for long time and thus is possible to make reliable tandem or series‐connected panels.

**Figure 4:**
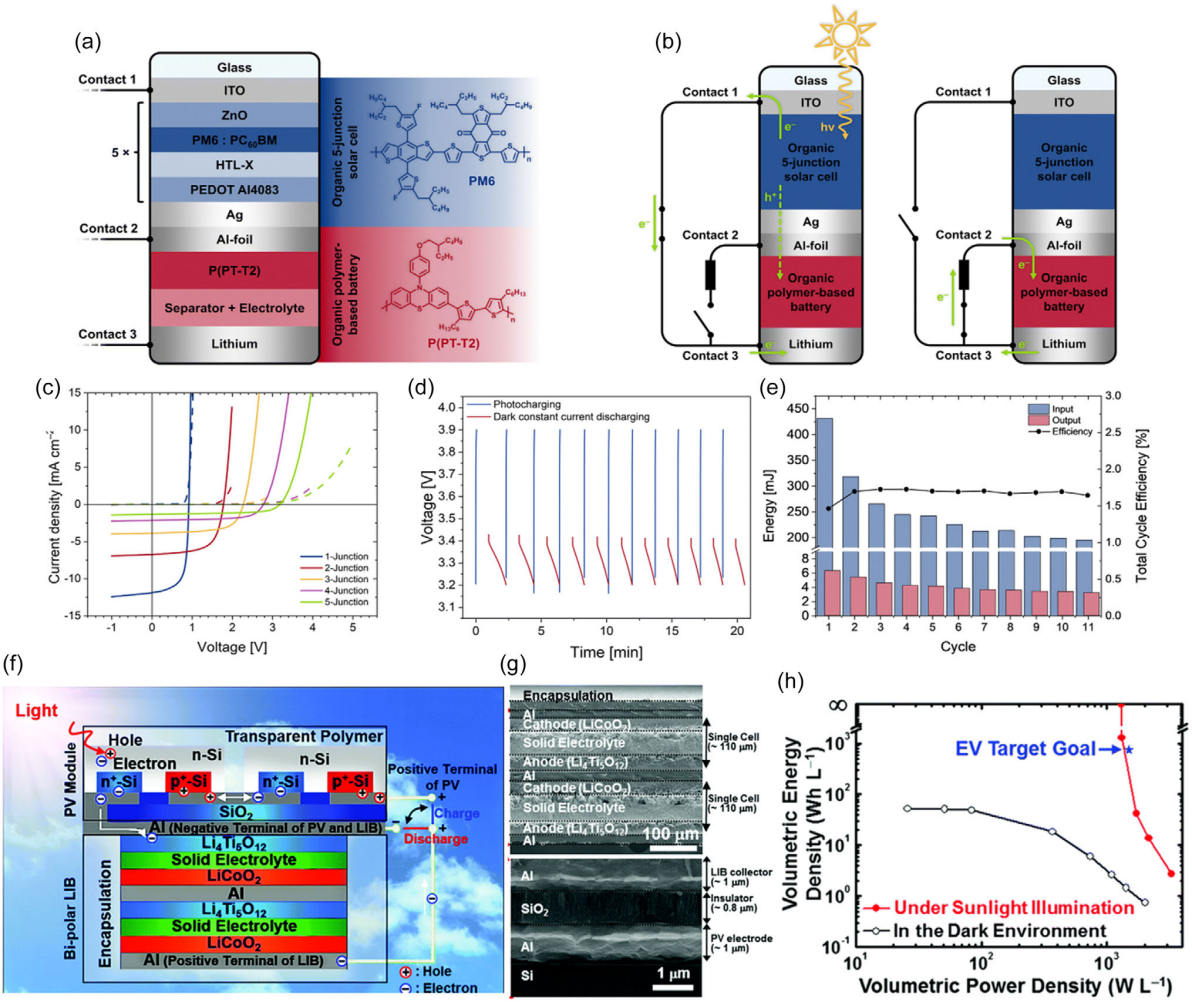
a) Schematic of the components of the 3t‐organic photo‐battery. b) Working principles of the device during photocharge and discharge in the dark. c) current‐voltage curves recorded for multiple solar cells with increasing number of junctions. d) Voltage profile of the photo‐battery during several cycles of photocharge and dark discharge with a fixed upper potential limit (3.9 V) and at a fixed discharge current of 0.7 C. e) Energy input during illumination, output during dark discharge, and resulting cycle efficiency. Reproduced with permission.^[^ ^47^ ^]^ Copyright 2023, Royal Society of Chemistry. f) Schematic representation of the internal structure and operating principle of the Si PV–LIB device under sunlight illumination. g) The seamlessly unitized interface between the bipolar LIB cell and c‐Si PV module, as shown in the scanning electron microscope. h) Ragone plots of Si PV–LIB devices under sunlight illumination (red line) and in the dark environment (black line). Reproduced with permission.^[^ ^48^ ^]^ Copyright 2017, Royal Society of Chemistry.

Würfel et al. developed a monolithic three‐terminal organic photo‐rechargeable battery, which combines a multijunction organic solar cell capable of charging up to 4.2 V under illumination conditions with an organic redox‐polymer‐based LIB (**Figure** **4**a).^[^ ^47^ ^]^ The five‐junction organic solar cell was prepared via solution process, which is composed of inverted structured absorber with PM6 (PBDB‐T‐2F) and PC60BM ([6,6]‐phenyl‐C61‐butyric acid methyl ester) acting as donor and acceptor respectively. To enhance the carrier's separation, ZnO particles and poly(3,4‐ethylenedioxythiophene) were applied to serve as the electron transport layer and hole transport layer respectively. The combined five‐junction organic solar cell was connected with an Al foil common current collector, which accepts the photogenerated holes and oxidizes the polymer electrode (P(PT‐T2)) of LIBs, and then the Li–ions from the electrolyte are reduced at the negative battery electrode by electrons originating from the negative electrode of the solar cell, resulting in the lithium deposition. A switch was applied to adjust the circuit structure and alter the photocharge and dark discharge processes as Figure 4b illustrates. The five‐junction organic solar cell exhibits high‐power conversion efficiency of 19%, successfully photocharging the battery without external bias at a quick response (Figure 4d). The device exhibits a discharge capacity of up to 22 mAh g^−1^ and an average discharge potential of 3.6 V and energy density of 69 Wh g^−1^. When discharged at dark, it delivers a total cycle efficiency of over 1%. These performances are attributed to the stable and high output voltage from organic solar cells (Figure 4c). The organic solar cells show advantages in their flexibility and lightweight; however, the long‐term stability remains a challenge to be addressed in the future. Especially under conditions with moisture, sunlight, or temperature variation, these factors might accelerate the decomposition of organic components.

The monolithic‐integrated 3t‐IPRB structure may vary according to the properties of PVs. Here is an example of an all‐solid‐state monolithic 3t‐IRPB with inorganic components reported by Lee et al.^[^ ^48^ ^]^ Specifically, the device is a monolithically integrated photo‐rechargeable power source combining 25 series‐connected miniaturized crystalline silicon (c‐Si) PVs, providing a high output voltage of 14.1 V and a power conversion efficiency of 15.8%, as illustrated in Figure 4f. The Al foil serves as an intermediate layer that being rear contact of the PV and the current collector of the Li_4_Ti_5_O_12_–LiCoO_2_ battery. What makes it different from the former work is that the common current collector Al foil layer accepts the photogenerated electrons rather than holes. The configuration sequence of 3t‐IPRBs is determined by the semiconductor properties of the PV, particularly the placement and characteristics of the p–n junction, whether it is at the front or rear contact. As presented in Figure 4g, the PV and energy storage materials seamlessly inkjet‐printed by sequence. The electron released from the c‐Si PV module reduces the anode: Li_4_Ti_5_O_12_ + xLi^+^ + x*e* ^−^ − Li_(4+x)_Ti_5_O_12_). Concurrently, free electrons are released from the positive terminal of the LIB cathode (LiCoO_2_ − Li_1‐x_CoO_2_ + xLi^+^ + x*e* ^−^) and then flow toward the positive terminal c‐Si PV module, resulting in electron recombination with the holes. The device exhibits an overall conversion‐storage efficiency of 7.61%, with a capacity retention rate of 98% after 100 cycles. Notably, as Figure 4h presented, numerous amounts of energy at power densities below 1000 W L^−1^ were produced by the device under sunlight illumination, which highlights its potential application in IPRB system.

### 3.2 Common Electrode 3t‐IPRBs

In this configuration, the common electrode not only provides a physical bridge between the photoelectrode and the energy storage electrode but also participates in redox chemistry. Unlike current collector‐based 3t‐IPRBs, where the intermediary layer does not participate in electrochemical reactions, a common electrode actively engages in redox processes—either directly through its own redox‐active sites or via interactions with redox shuttles in the electrolyte. Although it does not absorb photons, it mediates the interaction between photogenerated carriers and redox shuttles (e.g., I^−^/I_3_^−^ and S^2^^−^/S_x_^2^^−^) in the electrolyte, thereby reducing reaction overpotentials.

During charging, the photoelectrode and counter electrode are connected. Photogenerated electrons move from the photoelectrode to the counter electrode, while holes enter the electrolyte and oxidize redox shuttles (e.g., I^−^/I_3_^−^ or S^2^^−^/S_x_^2^^−^), stabilizing the potential in the system. During discharge, the photoelectrode is disconnected, and the common electrode functions as a cathode, guiding electrons from the energy storage electrode back to the external circuit and facilitating the oxidation of redox shuttles in the electrolyte.

A primary focus in common electrode design is minimizing reaction overpotentials by lowering activation barriers and mitigating polarization losses. This can be achieved through materials that offer catalytic surfaces, proper redox potential alignment with both the photoelectrode and storage electrode, and high conductivity combined with chemical stability. In many cases, composite architectures (e.g., carbon–metal hybrids, doped carbons) are employed to improve surface area, enhance conductivity, and resist corrosion or passivation.^[^ ^23^, ^49^, ^50^, ^51^ ^]^ By fine‐tuning the common electrode's electronic structure, catalytic activity, and robustness, 3t‐IPRBs can sustain low overpotentials and achieve stable, high‐power operation over extended cycling—demonstrating significant potential for efficient solar energy harvesting and storage.

Such a configuration is particularly suited for systems requiring multistep electron transfer reactions, such as lithium–sulfur (Li–S) batteries, lithium–oxygen (Li–O_2_) batteries, lithium–iodine (Li–I_2_) batteries, zinc–air batteries, and zinc–iodine (Zn–I_2_) batteries. Another type of 3t‐IPRBs design is the common electrode design. In this configuration, the common electrode actively participates in the reaction, either by directly reacting or by engaging with redox shuttles in the electrolyte, thereby being part of the energy storage system alongside the counter electrode. The photogeneration process could effectively accelerate the carrier's diffusion and reaction kinetics and are commonly applied in various systems especially multistep electron transfer reactions like Li–S batteries,^[^ ^50^ ^]^ Li–O_2_,^[^ ^52^ ^]^ Li–I_2_,^[^ ^53^ ^]^ Zn–air batteries,^[^ ^54^ ^]^ and Zn–I_2_ batteries.^[^ ^55^ ^]^ In most cases, the common electrode does not necessarily directly participate in the solar conversion process but acts as a mediator, connecting the electrolyte to form liquid semiconductor junction and reacting with the redox shuttle to regulate the potential.

**Figure 5:**
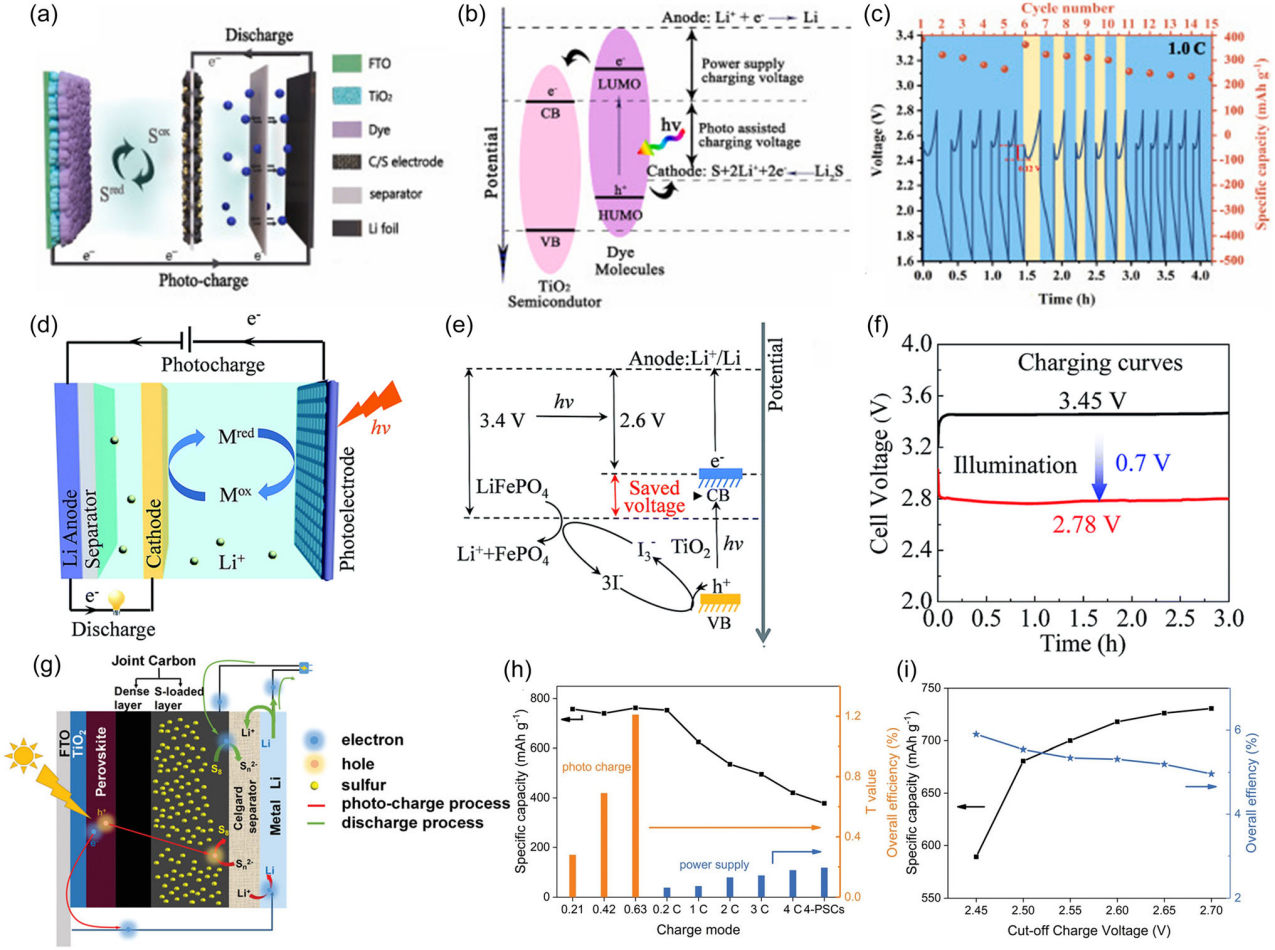
a) Device structure and b) energy diagram of the integrated 3t‐Li–S batteries. c) The cycling performance with alternating light‐assisted and dark charging of device at 1 C. Reproduced with permission.^[^ ^50^ ^]^ Copyright 2023, Elsevier. d) Schematic illustration of a photo‐assisted 3t‐LIB. e) The energy diagram of the photo‐rechargeable LIB. f) The charge curves of the photo‐assisted chargeable LIB (red line) and the LIB (black line) at a current density of 0.02 mA cm^−2^. Reproduced with permission.^[^ ^53^ ^]^ Copyright 2015, Royal Society of Chemistry. g) Schematic diagram of the 3t‐PSC–Li–S battery. h) Discharge capacities (black line) and T values (blue and yellow columns) of the battery under different charge modes. i) Discharge capacity and overall efficiency of different cutoff photocharge voltage. Reproduced with permission.^[^ ^23^ ^]^ Copyright 2019, Wiley‐VCH.

In the common electrode configuration, dye‐sensitized solar cells (DSSCs) and photoelectrochemical cells (PECs) demonstrate great potential to integrate with energy storage systems. DSSCs utilize organic dyes to absorb sunlight, generating electron‐hole pairs, with electrons injected into the semiconductor's conduction band and transferred to the battery for energy storage. A key component in DSSC‐based IPRB is the redox shuttle, such as iodide/triiodide (I^−^/I_3_^−^), which has a higher redox potential than the photoelectrode, allowing efficient electrons to transfer back to the dye. This process involves electron‐mediated reduction of I_3_^−^ to I^−^ at the counter electrode, sustaining dye regeneration and continuous photon absorption.^[^ ^52^ ^]^ The iodide/triiodide shuttle effectively reduces the energy barrier for the Li_2_O_2_ oxidation reaction and reduces the overpotential. Wu et al. demonstrated an example of photo‐assisted lithium–oxygen battery by integrating a dye‐sensitized TiO_2_ photoelectrode with an oxygen electrode via I^−^/I_3_^−^ redox shuttle. Upon illumination, the photoelectrode generates triiodide ions that diffuse to the oxygen electrode driving the oxidation of Li_2_O_2_, which significantly reduces the overpotential and reduces the charging voltage from above 4.0 to 3.6 V. The photovoltage generated by the TiO_2_ photoelectrode compensates for the high overpotential typically required during charging, enabling the device to operate under a negative overpotential regime. In addition to static batteries, photo‐assisted flow cells also exhibit significant promise for advanced energy storage applications. An aqueous Li−I solar flow battery integrated a built‐in dye‐sensitized TiO_2_ photoelectrode was demonstrated in 2015.^[^ ^56^ ^]^ LiI, guanidine thiocyanate, and saturated chenodeoxycholic acid have been selected as candidates for catholyte, which aims to provide redox mediator and surface–wettability. By utilizing the I^−^/I_3_^−^ redox couple, the photogenerated carriers efficiently drive the oxidation and reduction processes across separated compartments, with a ceramic lithium–ion conductive separator preventing electrolyte cross‐contamination. This system achieves a reduced charging voltage of 2.90 V under 1 sun illumination compared to the 3.30 V discharge voltage, yielding a 20% energy savings compared to conventional Li–I batteries. This work highlights the potential of a safe, cost‐effective, and energy‐efficient flow cell concept, demonstrating advancements in redox flow battery systems. Apart from the merits of 3t‐IPRBs, the dual‐electrolyte systems may cause interfacial cross‐contamination issues and low ionic conductivity, which could compromise the overall performance and stability of the integrated system. To address the issue, here as **Figure** **5**a illustrates, a mono‐electrolyte system was reported on a common electrode‐enabled photo‐assisted DSSC‐Li–S system.^[^ ^50^ ^]^ Lithium bis(trifluoromethanesulfonyl)azanide (LiTFSI)‐based electrolyte was applied, while the sulfur and multiwalled carbon nanotube (S/C) served as the common electrode. This work demonstrated a reduction in charging voltage by 0.12 V and an increase in discharge capacity up to 885 mAh g^−1^ under illumination, which resulted from the appropriate energy level design (Figure 5b). While the cycling stability of DSSC‐IPRBs needs to be further enhanced, the capacity degradation is obvious within the first 15 cycles (Figure 5c). In this work, the common electrode not only facilitates continuous redox shuttle regeneration but also ensures efficient coupling between the DSSC photovoltage and the battery voltage. Besides these photo‐assisted battery works, it is worth noting that the bias‐free photocharging batteries are enabled by DSSC. A recent study demonstrated that an unprecedented light‐to‐charge energy efficiency (*η*_overlal_) of 11.5% under dim light has been reported, attributed to rational design combining a dye‐sensitized TiO_2_ photoelectrode with redox mediators (Cu^+^/^2+^(dmp)_2_) and the LiMn_2_O_4_‐Li_2_Mn_2_O_4_ reaction.^[^ ^57^ ^]^ The cathodic reaction of LMO (≈+3.0 V vs. Li/Li^+^) has been selected to align the reduction potential of the storage electrode active material with the Fermi level of TiO_2_. This alignment is crucial for enabling a bias‐free photocharging process, as it ensures that the photogenerated electrons from the TiO_2_ conduction band can drive the reduction reaction at the storage electrode efficiently.

In summary, DSSC‐driven IPRBs achieve high open‐circuit voltages to trigger the charging process, while the solar conversion efficiency is still relatively low, and the photodegradation issues need to be addressed to ensure stability and longevity.

Differing from DSSC‐based 3t‐IPRBs, PEC‐based 3t‐IPRBs usually require a single electrolyte, which alleviates the electrolyte compatibility issues in DSSCs. It can even be built in an all‐solid‐state type. The PEC‐based 3t‐IPRB is composed of semiconductor photoelectrodes that directly generate electron‐hole pairs under illumination, driving electrochemical reactions at the electrode‐electrolyte interface. In addition, it is composed of a common electrode that streamlines charge transfer pathways and keeps the electron balanced during charge and discharge. These systems often use photo‐responsive materials like hematite and chalcogenides, focusing on direct solar‐to‐chemical energy conversion. Direct photo‐assisted charging reduces the overall system complexity and enhances efficiency. Zhou et al. reported an example of a PEC‐based IPRB, which integrates a photocatalytic TiO_2_ photoelectrode, an I^−^/I_3_^−^ redox shuttle, and a LiFePO_4_–Li battery, as shown in Figure 5d.^[^ ^53^ ^]^ The energy‐saving mechanism lies in the energy levels between electrodes and the semiconductor; specifically, the saved voltage is determined by the energy difference between the charging voltage of the LIB and the quasi‐Fermi level of electrons in the semiconductor (Figure 5e). The device achieved a photo charging voltage of 2.78 V, significantly lower than the discharging voltage of 3.41 V, resulting in ≈20% energy savings (Figure 5f). Yet this device is a photo‐assisted charging one that needs a bias to empower. By introducing tandem solar panels or series‐connected solar cells, the requirement for external power is eliminated. For instance, an integrated perovskite‐Li–S solar rechargeable battery was proposed by applying a common joint carbon electrode.^[^ ^23^ ^]^ Three perovskite solar cells (PSCs) were connected in series and generated a high open‐circuit voltage of 2.8 V to photocharge the Li–S battery without bias. PSCs generate electron‐hole pairs upon illumination, and then electrons move to the TiO_2_ layer, while holes move to the counter electrode (Figure 5g). As the crucial part of the PSC–Li–S system, the common carbonaceous electrode facilitates the formation and dissolution of lithium polysulfides and remains stable under prolonged cycling and continuous illumination, addressing common issues of degradation. As depicted in Figure 5i, the system achieves an overall energy conversion efficiency of 5.14%, maintaining a specific capacity of 750 mAh g^−1^ at a photocharge rate of 2 C.

All these studies highlight a similar underlying mechanism for the common electrode in 3t‐IPRB systems, emphasizing its unique role in facilitating efficient charge transfer, redox shuttle regeneration, and seamless integration between the photoelectrode and the energy storage components. To be more specific, during charging, the common electrode serves as a mediator for a two‐step redox process: Photogenerated electrons reduce the redox shuttle at the photoelectrode, which subsequently transfers these electrons to the common electrode. This ensures efficient electron transport to the battery anode while simultaneously supporting lithium–ion migration in the electrolyte. When discharging, the common electrode forms a closed circuit with the battery anode, enabling electron flow through the external circuit and sustaining ion migration in the electrolyte to complete the electrochemical cycle.

## 4 4t‐IPRBs

The 4t‐IPRBs typically consist of separate solar cells and energy storage components (rechargeable batteries), directly connected by wired interfaces to ensure efficient charge transfer and storage. The primary advantage of this setup lies in its separate configuration, which enables the ability to autonomously optimize both solar energy conversion and energy storage capability, thereby minimizing interdependent losses. From the thermodynamic point of view, 4t‐IPRBs maintain structural stability and longevity as no photoinduced phase transition occurs at the electrode, and the photoexcited carriers are directly injected into the energy storage part via an external circuit, which is the major characteristic different from the counterpart IPRBs.

Despite these advantages, 4t‐IPRBs face challenges in both the battery and solar cell components. Batteries often experience rate limitations due to sluggish ion transport and interfacial resistance, particularly under high photogenerated current densities, while long‐term cycling is hindered by structural degradation, electrolyte decomposition, and interface instability. Solar cells, constrained by the Shockley–Queisser limit, can degrade under ultraviolet exposure and thermal stress, reducing their efficiency and lifespan. Advanced solutions such as fabricating nanostructured electrodes, developing high‐entropy electrolytes, and designing tandem solar cells are promising for improving charge transport, stability, and photoconversion efficiency.^[^ ^58^, ^59^, ^60^, ^61^, ^62^, ^63^ ^]^

Apart from these factors, the interdependence between the PV module and the battery introduces complexities arising from dynamic external conditions, such as temperature and light intensity, and internal states like the SoC. The dynamic interaction between the PV module and the battery often results in mismatches in power transfer efficiency, as variations in environmental and operational conditions affect the performance of both components. Beyond static photovoltage considerations, dynamic matching of voltage and current is essential to maintain efficient energy transfer. As the SoC increases, the battery voltage rises necessitating higher photovoltage, while the concurrently reduced current creates additional challenges for energy alignment. In addition, high‐charge current densities further exacerbate overpotential, decreasing overall energy efficiency. Strategies such as incorporating MPPT electronics, applying electrode coatings, engineering heterostructured materials, and optimizing electrolytes enhance system performance by improving carrier transport and mitigating impedance mismatches. While similar issues arise in other IPRB configurations, such as 2t‐IPRBs and 3t‐IPRBs, the primary sources of energy loss in those systems differ, as outlined earlier in the discussion.

Architecturally, the 4t configuration is formed by directly connecting the cathode and anode of the solar cell to the corresponding terminals of the battery. Single‐junction solar cells usually deliver open‐circuit voltage under 1 V; for chalcogenide thin‐film solar cells, the value ranges from 0.5 to 0.9 V, while 0.5 to 1.0 V for organic PV (OPV) cells and 0.9 to 1.2 V for perovskite solar cells. To meet the charging voltage requirements of alkali–ion rechargeable batteries, solar cells are often connected in series. This configuration ensures that the combined output voltage is sufficient for effective charging. However, integrating solar panels with rechargeable batteries involves more than just achieving a voltage match; it also requires careful consideration of the dynamic interaction between the PV module and the battery during operation. This interaction is closely tied to the battery's SoC, which influences several thermodynamic and operational factors. As the SoC changes, the battery's voltage and impedance vary, affecting how efficiently the PV module can transfer power. For instance, as the battery charges and its voltage increases, the PV module must adjust to maintain effective power transfer. This dynamic relationship is crucial in minimizing energy loss and optimizing overall system efficiency. Thermodynamic behaviors, such as the battery's heat generation and entropy changes, are also influenced by the SoC and play a significant role in system performance. The PV module's ability to match its output with the battery's varying requirements, particularly under fluctuating environmental conditions like temperature and light intensity, is essential for maintaining optimal energy conversion and storage. Understanding these thermodynamic interactions and the real‐time dynamics between the PV and battery components is critical for reducing inefficiencies and enhancing the effectiveness of the integrated system.

In 4t‐IPRBs, the solar panel serves as the current source (dependent on light intensity), while the battery plays the role of a load with variable resistance (dependent on SoC). The MPP of solar cell is a crucial parameter, as it ensures the condition where maximum power is delivered to the load. However, due to variations in load resistance, the solar cell often operates away from the MPP, resulting in reduced power output. Traditional approaches use a MPPT inverter to optimize power output. However, MPPT systems add complexity, extra resistance, cost, and energy overhead. Directly connecting PV modules to batteries, without intermediary power management elements, has been proposed as a cost‐effective alternative to traditional MPPT systems. This approach leverages the natural alignment of the PV module's MPP with the battery's operating range, potentially simplifying system design and reducing costs.

A work from Merdzhanova et al. explored the feasibility of directly coupling battery with PV. In their work, a seven‐cell silicon heterojunction modules are incorporated with a 160 mAh Li–ion battery.^[^ ^64^ ^]^ The system maintained a high coupling factor, consistently above 90%, across a wide range of irradiance levels (0.02 to 1 Sun) and temperatures (25–70 °C). While fluctuations were observed due to changes in the battery's SoC, the overall efficiency remained stable. They concluded that the battery played the role of buffer, stabilizing and balancing the power, thereby ensuring persistent coupling efficiency even when the load varied. This work demonstrated comparable performance and robustness of 4t‐IPRBs to MPPT systems, especially in terms of peak efficiency under practical environmental conditions with variable irradiance and temperature. However, the mechanism by which battery dynamics as a buffer in 4t‐IPRBs remains unclear. Specifically, the impact of battery dynamics response to PV and the reciprocal response from the PV on the battery needs further investigation.

Another work from Astakhov also measured the coupled PV‐battery module, which achieved an average coupling factor of over 99.8%.^[^ ^65^ ^]^ The power losses in 4t‐IPRB system were analyzed, including the dark current loss and internal resistance in wiring and battery. The wiring loss could be treated as an extrinsic factor, while the dark current is considered an intrinsic factor raised from defects, impurities, and inherent semiconductor characteristics from PV. Both studies underscore the significant potential of 4t‐IPRBs as next‐generation solutions for efficient solar energy storage. While these works emphasize overall system performance, including coupling efficiency and operational stability, their focus is primarily on system‐level metrics rather than the intrinsic dynamics of the battery itself. The battery impedance keeps changing with the SoC, making it impossible to consider it as a constant resistance component. In 4t‐IPRBs, the charging current is influenced by the load impedance of the PV, resulting in electrical behavior that deviates significantly from a stable, galvanostatic condition. Additionally, battery polarization substantially affects the electrochemical behavior, complicating the task of maintaining power alignment with the solar cells. These intrinsic variations are further influenced by the choice of electrode materials, electrolytes, and the overall cell structure.

**Figure 6:**
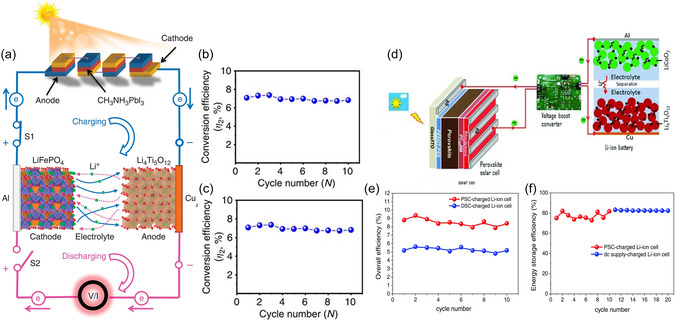
a) Schematic diagram of the fabricated system of PSC–LIB. b) Overall photoelectric conversion efficiency of the PSCs–LIB device. c) Energy storage efficiency of LIB. Reproduced with permission.^[^ ^66^ ^]^ Copyright 2015, Springer Nature. d) Photocharging diagram of Li_4_Ti_5_O_12_–LiCoO_2_ cell using PSC. e) Overall efficiency of the PSC and dye sentitized solar cell‐charged Li_4_Ti_5_O_12_‐LiCoO_2_ cell. f) Energy storage efficiency of PSC‐charged Li_4_Ti_5_O_12_–LiCoO_2_ cell. Reproduced with permission.^[^ ^67^ ^]^ Copyright 2017, Wiley‐VCH.

The study by Xu et al. demonstrates the direct photocharging of LIBs using series‐connected PSCs.^[^ ^66^ ^]^ As demonstrated in **Figure** **6**a, the system integrates four single‐junction CH_3_NH_3_PbI_3_‐based PSCs to directly charge LIBs constituted by a LiFePO_4_ cathode and a Li_4_Ti_5_O_1__2_ anode. This configuration demonstrated a V_oc_ of 3.84 V under 1 sun, slightly above the charging cutoff voltage of LiFePO_4_‐Li_4_Ti_5_O_12_ LIBs (2.5 V) to trigger the charging. The power conversion efficiency is 12.65% for the connected PSC (perovskite solar cell) units, and overall photoelectric conversion and storage efficiency yield 7.8% (Figure 6b), while the battery shows little degradation within 15 cycles. As can be seen from Figure 6c, the LIBs exhibit insufficient energy storage efficiency which remains lower than 70%. It is noted that there is substantial energy loss (≈38.3%) after integration, which could be attributed to the voltage mismatch and the overpotential from LIBs.

To address the power mismatches issues and reduce energy losses, a work introduced a DC–DC converter to match the solar cell output and the battery charging requirements.^[^ ^67^ ^]^ In this work, single‐junction solar cell was directly incorporated with DC–DC converter to lift its output voltage then charge the battery, as presented in Figure 6d. The p‐i‐n CH_3_NH_3_PbI_3_‐based PSC and DSSC are used as power sources, yielding a conversion efficiency of 14.2 and 7.89% respectively. The LiCoO_2_‐Li_4_Ti_5_O_12_ LIB demonstrated low overpotentials during cycles, which is essential for energy retrieval. Instead of connecting solar cells in series to meet the battery charging voltage, the DC–DC converter was applied to adjust the output of the single‐junction solar cell. The converter‐implemented PV–battery system shows an enhanced overall efficiency of 9.36% for a PSC power source (Figure 6e), which shows a reduction in energy loss (≈34.1%). The Li_4_Ti_5_O_12_‐LiCoO_2_ cell witnessed a slight increase in energy storage efficiency (Figure 6f), which is a crucial part of high overall efficiency. It can be inferred that the corporation of the converter in 4t‐IPRBs is a stopgap solution that does not fully address the underlying energy loss issue. The intrinsic factor in the battery including polarization change may affect the response from solar cells, causing the mismatch during integration.

Furthermore, unlike traditional 2t or 3t configurations, 4t‐IPRBs decouple the energy harvesting and storage processes, thereby enabling superior energy management and operational efficiency. Besides, it holds the promise of high flexibility when coupling the thin film solar cell with flexible energy storage components. This architectural versatility is crucial for applications in wearable technology, where devices must seamlessly integrate into dynamic and changing environments while maintaining consistent performance under mechanical deformation. Recent advancements in material science, particularly the integration of high‐efficiency PSCs and flexible ZIBs, have further enhanced the potential of 4t‐IPRBs for use in wearable electronics and portable devices. These integrations not only provide robust energy storage and conversion capabilities but also support the development of lightweight, flexible systems that are well‐suited for the next generation of wearable and portable electronics.

**Figure 7:**
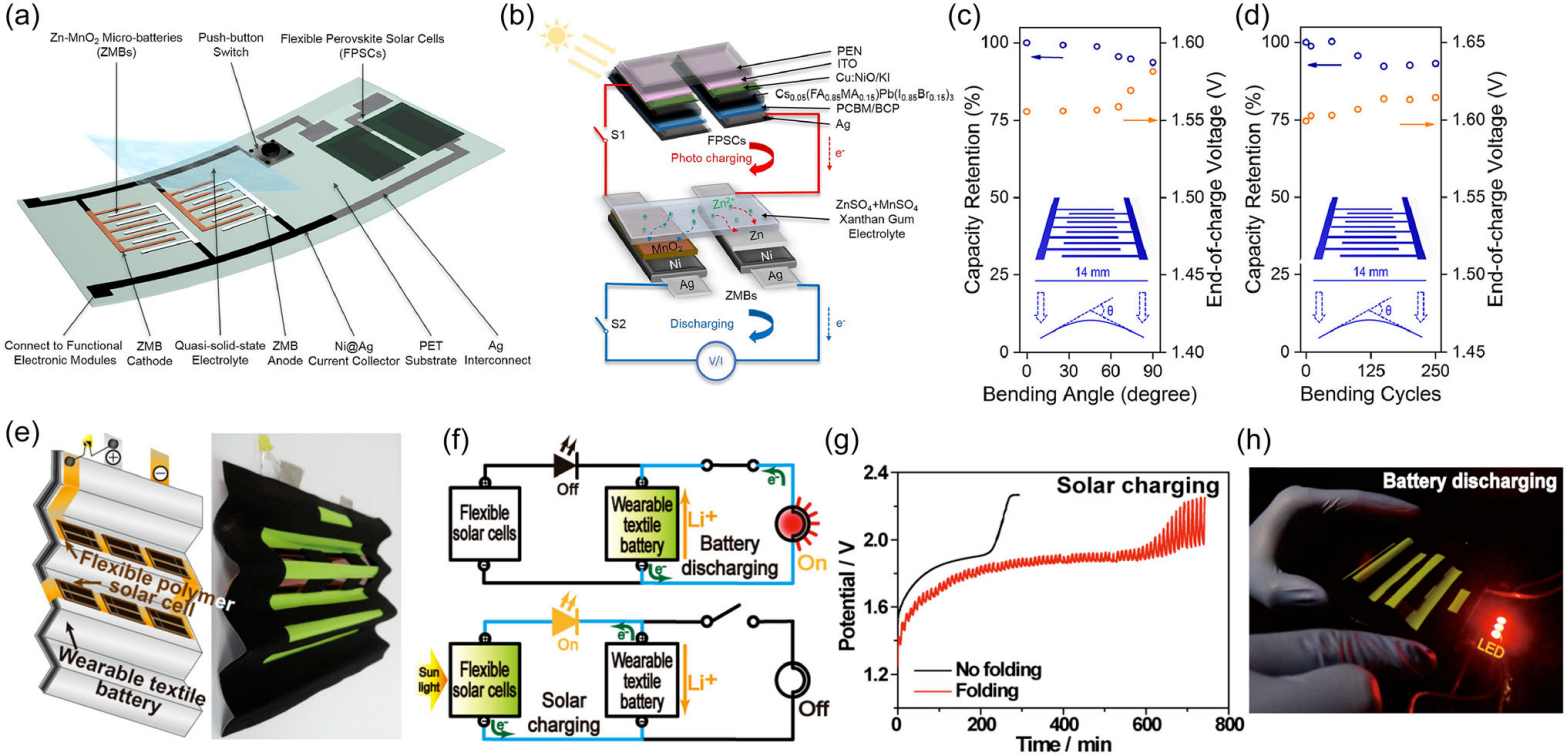
Schematics of the a) device configuration and b) working principle of the integrated flexible photo‐rechargeable system. c) Capacity retention and end‐of‐charge voltage of Zn–MnO_2_ battery (ZMB) at different bending angles. d) Capacity retention and end‐of‐charge voltage of ZMB after repeated bending cycles from 0° to 65°. Reproduced with permission.^[^ ^68^ ^]^ Copyright 2022, Elsevier. e) Schematic representation and photograph of the textile battery integrated with polymer solar cells. f) Equivalent circuits of a solar rechargeable textile battery in the discharging and solar‐charging modes. In the discharging mode, the battery turns on light bulbs, and, in the solar‐charging mode, the battery is charged, which is indicated by an LED. g) Potential profiles of the textile battery during solar charging in the presence and absence of repeated folding–unfolding motions. Each folding–unfolding cycle takes 10 min. h) A demonstration of battery operation. Reproduced with permission.^[^ ^69^ ^]^ Copyright 2013, American Chemical Society.

Building on these advancements, a flexible and wearable 4t‐IPRB was developed by incorporating quasi‐solid‐state ZIBs with PSCs.^[^ ^68^ ^]^ Energy storage parts (ZIBs) and energy harness parts (PSCs) are connected by an inkjet‐printed Ag/Ni current collector on a polyethylene terephthalate substrate. The two ZIBs are connected by current collector, each of them composed of Zn–MnO_2_ interdigitated electrodes by inkjet printing method (**Figure** **7**a). A quasi‐solid‐state gel is applied to provide mechanical stability and flexibility and enables ion transport. As depicted in Figure 7b, the power harness part is formed by two series‐connected PSCs, with a spin‐coated passivation layer (choline chloride), absorber (Cs_0.05_(FA_0.85_MA_0.15_)Pb(I_0.85_Br_0.15_)_3_), and an electron transport layer (PC_61_BM). The series‐connected PSCs exhibit a conversion efficiency of 11.1%. At the discharge current of ≈22, ≈56, and ≈167 C, the system illustrates overall efficiency of 5.28, 5.25, and 4.95%, respectively. The flexible substrate and materials ensure its working under a bending condition (Figure 7c,d).

Lee et al. introduced a foldable solar‐rechargeable battery incorporating textile LIBs and a flexible organic polymer solar cell (Figure 7e).^[^ ^69^ ^]^ This research developed functional battery components including the current collector (textile matrix), binder, and separator, to integrate seamlessly with solar cells and create durable devices capable of withstanding extensive folding. A textile Ni‐coated polyester was implemented as a robust matrix within the flexible device. Additionally, a polyurethane‐based binder and separator were applied to facilitate the adhesion of electrodes (Li_4_Ti_5_O_12_ and LiFePO_4_) to the current collectors and the wettability. Apart from these optimizations, the flexible solar cells were fabricated on an indium tin oxide‐coated polyethylene naphthalate substrate, achieving a conversion efficiency of 5.49%. As presented in Figure 7g,h, the device was tested under original and folded conditions, and it is capable of powering a light‐emitting diode (LED).

In conclusion, though the complexity of the material design and the need for precise integration present challenges in manufacturing and scalability, it offers several significant advantages, including enhanced flexibility and reliability under mechanical stress. These attributes mark a stride in wearable and portable self‐powered energy storage solutions. By integrating the quasi‐solid‐state batteries with high‐efficiency thin‐film PV techniques, the 4t‐IPRBs achieve a remarkable balance between flexibility and efficiency, paving the way for future self‐powered portable energy applications.

Loss is a general and prevalent issue in 4t‐IPRB systems regardless of what the integration techniques are applied. The primary cause is mismatches arising from intrinsic factors rather than extrinsic integration factors. Here, we classified the energy loss into two types: the extrinsic mismatch caused by wiring and integration techniques and the intrinsic factors arising from materials, including the dark current from PV and battery polarization. It is worth noting that the intrinsic loss is not simply the sum of each component's individual contributions but arises from their dynamic interactions. In 4t‐IPRB simulation works, the battery is often modeled using a Randles circuit with a zero‐time constant, allowing its current‐voltage (I–V) characteristics to be expressed as a linear function dependent solely on the SoC. However, practical rechargeable batteries exhibit nonlinear I–V characteristics due to polarization, phase transitions, and overpotential. The impedance in rechargeable batteries fluctuates, influencing the I–V characteristics and causing shifts in the PV's operating point. Consequently, the PV current deviates from a steady state, leading to varying electrochemical behaviors during battery polarization. Another critical concern is the deviation of the working point of the battery from the MPP of the PV module, especially when the battery approaches full charge. As when the battery approaches a fully charged state, the ion diffusion of lithium ions is hindered by the saturated active sites, resulting in decreased current flow.^[^ ^70^ ^]^ This creates a power mismatch where the PV module generates more power than the battery can accept, forcing the system to deviate from the MPP. Additionally, the charging dynamics on the battery side, including solid‐electrolyte interphase (SEI) growth and overpotential buildup, further contribute to energy losses.

When working under constant illumination, the thermal effects could pose a significant challenge in stability of non‐aqueous IPRBs, particularly impacting the SEI. The SEI, typically formed by the decomposition of electrolyte components at the electrode surface during initial cycling, acts as a passivation layer that prevents further electrolyte decomposition while allowing ion transport. However, the thermal stress promotes additional decomposition of the SEI, generating gaseous species byproducts and compromising its protective properties.^[^ ^71^ ^]^ This degradation increases the interfacial impedance, reducing reversibility, lithium–ion transport efficiency, and exacerbating capacity fade during cycling.^[^ ^72^ ^]^ To mitigate the degradation, significant efforts have been made to improve the thermal stability of the SEI by electrolyte engineering including applying fluorinated solvents and adding additives.^[^ ^58^, ^73^ ^]^

For long‐term operations, the aging of both components is also a challenge to be addressed. Battery loses active materials (Li/Na/Zn) after cycles, leading to capacity degradation, and the PV's lifespan may reduce under high temperatures. These issues need to be addressed by rational material optimization on both sides.

To sum up, though the PV–battery combination exhibits great potential in next‐generation self‐charging systems, further application is hindered by substantial integration energy loss. Besides focusing on the systematic construction and the measurement of photocharging, the understanding of intrinsic variations and their impacts requires further investigation. In this part, we state the key sources of these losses and explore potential strategies for mitigating them, aiming to provide pathways for improving the performance and efficiency of IPRBs.

## 5 Conclusion

To date, the research on IPRBs still remains in its nascent stage. Current emerging research emphasizes the feasibility of developing innovative materials and novel device architectures, which offer great potential for high‐performance systems, yet achieving the optimal combination of materials remains a challenge. There are significant issues that need to be addressed before achieving real‐world practical application, such as insufficient efficiency, limited longevity, material compatibility issues, and fabrication complexity. Despite these hurdles, IPRBs remain a very promising choice for next‐generation self‐powered energy storage solutions in scenarios where electricity is absent or insufficient. These issues can only be addressed by gaining a deeper understanding of the working principles, electrochemical signature, and energy loss mechanisms before they pave the way for industrial and commercial applications.

In this review, we revisited the previous works on this ascendant field and classified them into three major types according to their working principle, structural characteristics and electrochemistry. Besides, we unveiled the energy loss mechanism in each system and provided insights for the design and fabrication of high‐performance IPRBs.

The 2t‐IPRBs offer several advantages, including the most compact design and simplified fabrication process making it suitable for various applications. However, this configuration also presents less comparative efficiency with its counterpart IPRBs. The dual‐functional nature of the photoelectrode leads to higher rates of electron‐hole recombination and energy losses due to mismatched energy levels between electrodes and electrolytes interfacial. Moreover, there is an intrinsic thermodynamic mismatch between the lifetimes of photoexcited charges and the surficial redox reactions, resulting in a significant waste of charge carriers. Another concern is the photo corrosion of dual‐functional electrodes when exposed to electrolytes, which impedes long‐term cycling stability. Additionally, the conductive materials and binders used in the electrode block and shield sunlight from reaching the absorber, thereby further reducing the overall efficiency. Future research should focus on developing heterostructure electrodes and developing electrolytes with well‐matched energy levels to reduce carrier recombination. Besides, creating optimized binder and conductive systems can help minimize light loss. Furthermore, developing micronanostructures can accelerate electrode reaction kinetics.

The 3t‐IPRBs alleviate some of the issues in 2t‐IPRBs, as the photoelectrode could be isolated from the electrolyte thus eliminating the photo corrosion. In the common current collector design, the photoelectrode does not necessarily participate in the photo(de)intercalation or phase transition reaction, which improves stability and enhances carrier separation. In the common electrode 3t‐IPRB, each electrode maintains direct contact with the electrolyte. The photoelectrode drives a redox shuttle process triggered by photogenerated electrons, instead of undergoing direct photoinduced phase transitions. By serving as an intermediary, the common electrode decouples the photoelectrode from direct photo(de)intercalation with the electrolyte, which significantly enhances system stability and optimizes charge separation. The primary issue with three‐terminal systems is the energy loss caused by mismatched energy levels at various interfaces. These include the contact between the common electrode and the electrolyte, as well as the contact between the electrodes and the current collectors. In addition, the fabrication process is more complicated compared with its counterparts IPRBs; it requires a high‐standard encapsulation process to prevent electrolyte leakage. To address these challenges, future work could focus on aligning energy levels through material engineering and surface modification, optimizing interfaces with ultrathin interfacial layers via precise techniques like atomic layer deposition, and developing high‐conductivity solid electrolytes.

The 4t‐IPRBs, with their standalone operation principle on PV cells and rechargeable batteries, offer most competitive performance in terms of efficiency, stability, and design flexibility. Although this configuration sacrifices integration compactness, it provides the highest efficiency and stability, making it the closest to real‐world application among the three types. The primary issues in 4t‐IPRBs stem from a lack of understanding regarding the effects of the photocharging posed on the battery. Unlike the galvanostatic mode, the battery's response to the photocharging current leads to unpredictable polarization and changes in impedance. The impedance variations, in turn, affect the characteristics of the PV cells, resulting in significant energy losses due to intrinsic dynamic mismatches. Additionally, energy losses during the integration process, including wiring losses, further exacerbate the overall energy loss in 4t‐IPRBs. Hence, future work could focus on detailed studies to understand the effects of photocharging on battery performance and impedance changes and develop advanced integration technologies that ensure low resistance and high reliability.

In conclusion, each type of IPRB presents distinct advantages and challenges, making them suitable for different application scenarios. For instance, solar power supply is suitable for self‐powered wireless portable devices, such as smart electronics/optoelectronics, wearable sensors, and IoT, where compact integration of energy harness and storage is crucial.^[^ ^22^, ^53^, ^74^, ^75^, ^76^, ^77^ ^]^ In larger‐scale fields such as electric vehicles and building‐integrated energy systems, IPRBs can also be incorporated into rooftop designs, providing dual functionality for energy conversion and storage while conserving valuable space.^[^ ^78^ ^]^ Furthermore, their compact size and lightweight nature make IPRBs highly promising for aerospace applications, offering self‐sufficient energy solutions for long duration.^[^ ^79^, ^80^, ^81^ ^]^ Moving forward, research should focus on addressing the challenges on material and system to fully unlock their potential. By overcoming these obstacles and leveraging their unique strengths, IPRBs stand as a transformative technology for next‐generation sustainable, high‐performance, and self‐powered energy systems.

## Conflict of Interest

The authors declare no conflict of interest.

## Author Contributions

**Tianyun Qiu**: conceptualization (lead); writing—original draft (lead); and writing—review and editing (lead). **Wei Zhang**: writing—review and editing (supporting). **Xiaojing Hao**: writing—review and editing (supporting). **Kaiwen Sun**: writing—review and editing (supporting).
